# Gene tree parsimony for incomplete gene trees: addressing true biological loss

**DOI:** 10.1186/s13015-017-0120-1

**Published:** 2018-01-19

**Authors:** Md Shamsuzzoha Bayzid, Tandy Warnow

**Affiliations:** 1 Department of Computer Science and Engineering, Bangladesh University of Engineering and Technology, Dhaka, Bangladesh; 2 Department of Computer Science, University of Illinois Urbana-Champaign, Urbana, USA

**Keywords:** Gene duplication and loss, Gene tree parsimony, Deep coalescence, Dynamic programming

## Abstract

**Motivation:**

Species tree estimation from gene trees can be complicated by gene duplication and loss, and “gene tree parsimony” (GTP) is one approach for estimating species trees from multiple gene trees. In its standard formulation, the objective is to find a species tree that minimizes the total number of gene duplications and losses with respect to the input set of gene trees. Although much is known about GTP, little is known about how to treat inputs containing some *incomplete gene trees* (i.e., gene trees lacking one or more of the species).

**Results:**

We present new theory for GTP considering whether the incompleteness is due to gene birth and death (i.e., true biological loss) or taxon sampling, and present dynamic programming algorithms that can be used for an exact but exponential time solution for small numbers of taxa, or as a heuristic for larger numbers of taxa. We also prove that the “standard” calculations for duplications and losses exactly solve GTP when incompleteness results from taxon sampling, although they can be incorrect when incompleteness results from true biological loss. The software for the DP algorithm is freely available as open source code at https://github.com/smirarab/DynaDup.

## Background

The estimation of species trees is often performed by estimating multiple sequence alignments for some collection of genes, concatenating these alignments into one supermatrix, and then estimating a tree (often using maximum likelihood or a Bayesian technique) on the resultant supermatrix. However, this approach cannot be used when the species’ genomes contain multiple copies of some gene, which can result from gene duplication. Since gene duplication and loss is a common phenomenon, the estimation of species trees requires a different type of approach in this case.

The most powerful approaches for species tree estimation for multi-copy gene families are likely to be methods such as Phyldog [1], which co-estimate gene trees and species trees under parametric models of gene evolution that include duplications and losses. Another type of approach uses initial assignments of orthology and paralogy to inform gene tree and species tree estimation [2]. However, by far the most common approach for estimating species trees uses gene tree parsimony, which we now describe.

Gene tree parsimony (GTP) is an optimization problem for estimating species trees from a set of gene trees (estimated from individual gene sequence alignments). In its most typical formulations, only gene duplication and loss are considered, so that GTP depends upon two parameters: $c_d$ (the cost for a duplication) and $c_l$ (the cost for a loss). The two most popular versions of GTP are MGD (minimize gene duplication), for which $c_d=1$ and $c_l=0$, and MGDL (minimize gene duplication and loss), for which $c_d=c_l=1$. The version of GTP that seeks the tree minimizing the total number of losses has also been studied; for this, $c_d=0$ and $c_l=1$. These variants of GTP are NP-hard optimization problems [3], but software such as DupTree [4] and iGTP [5] for GTP are in wide use.

Basic to all these problems is the ability to compute the number of duplications and losses implied by a species tree and gene tree. This problem is called the “reconciliation problem”, surveyed in [6], and intensively studied in the literature (see, for example, [3, 7–17]). The mathematical formulation of the reconciliation problem was derived for the case where the gene tree and the species tree have the same set of taxa, and then extended to be able to be used on *incomplete* gene trees, i.e., trees that can miss some taxa.

Incomplete gene trees are quite common, and can arise for two different reasons: (1) *taxon sampling*: the gene may be available in the species’ genome, but was not included for some reason in the dataset for that gene, or (2) *gene birth/death*: as a result of gene birth and death (true biological gene loss), the species does not have the gene in its genome.

Given a gene tree *gt* and a species tree *ST*, two formulations for the number of losses have been defined. The most commonly used one computes the number of losses by first computing the “homeomorphic subtree” *ST*(*gt*) of *ST* induced by *gt*, and then computing the number of losses required to reconcile *gt* with *ST*(*gt*) (see, for example, [3, 8, 17]). Although this second formulation is in wide use (and is the basis of both iGTP [5] and Duptree [4], two popular methods for “solving” GTP), we will show that this can be incorrect when incompleteness is due to true biological loss. We refer to this formulation as the “standard” approach because of this widespread use in both software and the theoretical literature on GTP. The other, described in [18, 19], correctly computes the number of losses when incompleteness is a result of true gene loss, as we will prove.

This paper addresses the GTP problem for the case where some of the input gene trees may be incomplete due to either sampling or true biological loss. The main results are as follows:

- We formalize the duploss reconciliation problem when gene trees are incomplete due to taxon sampling as the “optimal completion of a gene tree”, and we prove (Theorem 1) that the standard calculation correctly computes losses for this case.
- We show by example that the standard calculation for losses in GTP can be incorrect when incompleteness is due to true biological loss.
- We show how to compute the number of losses implied by a gene tree and species tree, when incompleteness is due to true biological loss.
- We formulate variants of the GTP problem (when gene tree incompleteness is due to true biological loss) as minimum weight maximum clique problems (see Theorem 11 for one duploss variant), and show how to solve these problems efficiently using dynamic programming. We show that these optimal cliques can be found in polynomial time in the number of vertices of the graph, because of the special structure of the graphs. We also show that a constrained version of these problems, where the subtree-bipartitions of the species tree are drawn from the subtree-bipartitions of the input gene trees, can be solved in time that is polynomial in the number of gene trees and taxa.

## Basics

### Notation and terminology

We now define some general terminology we will use throughout this paper; other terminology will be introduced as needed. Throughout this paper we will assume that gene trees and species trees are rooted binary trees, with leaves drawn from the set $\mathcal {X}$ of *n* taxa, and we allow the gene trees to have multiple copies of the taxa, and even to miss some taxa. We orient each tree so that the root is on top and the leaves are at the bottom; hence, we also say that a node *v* is above node *w* if the path from *w* to the root of the tree goes through the node *v* (similarly we say that *w* is below *v*).

We let *gt* denote a gene tree and *ST* denote a species tree. We let *L*(*t*) denote the set of taxa at the leaves of the tree *t*, and require that $L(gt) \subseteq L(ST)$. If $L(gt) = L(ST)$ we say that *gt* is complete, and otherwise we say that *gt* is incomplete.

Let *T* be a rooted binary tree. We denote the set of vertices of *T* by *V*(*T*), the set of edges of *T* by *E*(*T*), the root by *r*(*T*), the internal nodes by $V_{int}(T)$, and the set of taxa that appear at the leaves by *L*(*T*). Note that if *T* is a gene tree, it can be incomplete, and so it is possible for |*L*(*T*)| to be smaller than the number of leaves in *T*. A *clade* in *T* is a subtree of *T* rooted at a node in *T*, and the set of leaves of the clade is called a *cluster*. Given a node *v* in *T*, the cluster of leaves below *v* is denoted by $c_T(v)$, and the subtree of *T* rooted at *v* is denoted by $T_v$. The most recent common ancestor (MRCA) of a set *A* of leaves in *T* is denoted by $MRCA_T(A)$. Given a gene tree *gt* and a species tree *ST*, we define $\mathcal {M}: V(gt) \rightarrow V(ST)$ by $\mathcal {M}(v) = MRCA_{ST}(c_{gt}(v))$. Finally, given a node *u* in a rooted binary tree, we let *r* denote the right child of *u* and *l* denote the left child of *u*.

For a rooted gene tree *gt* and a rooted species tree *ST*, where $L(gt)\subseteq L(ST)$, an internal node *v* in *gt* is called a duplication node if $\mathcal {M}(v) = \mathcal {M}(v')$ for some child $v'$ of *v*, and otherwise *v* is a speciation node [3, 8, 17, 20].

*ST*(*gt*) is the homeomorphic subtree of *ST* induced by the taxon set of *gt*, and is produced as follows: *ST* is restricted to the taxon set of *gt*, and then nodes with in-degree and out-degree 1 are suppressed. $ST^*(gt)$ is the tree obtained by restricting *ST* to the taxon set of *gt*, but not suppressing nodes of in-degree and out-degree 1.

We say that clade *cl* in *ST* is a *missing clade* with respect to *gt* if $L(gt) \cap L(cl) = \emptyset$, and a *maximal missing clade* if it is not contained in any other missing clade. Maximal missing clades that are descendants of $\mathcal {M}(r(gt))$ are called the “lower” maximal missing clades, and those that are not descendants of $\mathcal {M}(r(gt))$ are called the “upper” maximal missing clades. We denote by *LMMC*(*gt*, *ST*) (or *LMMC*), the set of lower maximal missing clades, and *UMMC*(*gt*, *ST*) (or *UMMC*), the set of upper maximal missing clades. Note $UMMC(gt,ST) = \emptyset$ if $\mathcal {M}(r(gt))=r(ST)$.

### The standard formula for computing losses

The *standard* formula (see, for example, [3, 8, 16, 17, 20]) for computing the minimum number of losses of a (potentially incomplete) gene tree *gt* with respect to a species tree *ST* is denoted $L_{std}(gt,ST)$, and is defined to be $L_{std}(gt,ST) = \sum _{u \in V_{int}(gt)} F(u,ST(gt)),$ where *F*(*u*, *T*) is defined for internal nodes *u* with children *l* and *r* (which can be interchanged in the formula below) by:

1 $$\begin{aligned} F(u,T) = \left\{ \begin{array}{lll} d(\mathcal {M}(r), \mathcal {M}(u)) + 1 &{} \text {if}\, \mathcal {M}(r) \ne \mathcal {M}(u)~ \& ~\mathcal {M}(l) = \mathcal {M}(u),\\ d(\mathcal {M}(l), \mathcal {M}(u)) + 1 &{} \text {if}\, \mathcal {M}(l) \ne \mathcal {M}(u)~ \& ~\mathcal {M}(r) = \mathcal {M}(u),\\ d(\mathcal {M}(r), \mathcal {M}(u)) \\ + d(\mathcal {M}(l),\mathcal {M}(u))&{} \text {if}\,\mathcal {M}(r) \ne \mathcal {M}(u)~ \& ~\mathcal {M}(l) \ne \mathcal {M}(u),\\ 0 &{} \text {if}\, \mathcal {M}(r)=\mathcal {M}(l)=\mathcal {M}(u). \end{array} \right. \end{aligned}$$

where $d(s,s')$ is the number of internal nodes in *T* on the path from *s* to $s'$. When *gt* is complete, then $ST(gt)=ST$, and this formula follows from [18].

*Optimal completion of a gene tree:*

- *Input* rooted binary gene tree *gt* and rooted binary species tree with $L(gt) \subseteq L(ST)$.
- *Output* complete gene tree $T_{samp}(gt,ST)$ that is an extension of *gt* such that $T_{samp}(gt,ST)$ implies a minimum number of losses with respect to *ST*.

In other words, we add all the missing taxa into *gt* (each taxon added at least once, but perhaps several times) so as to produce a complete binary gene tree that has a minimum number of losses with respect to *ST*. Let $L_{samp}(gt,ST) = L_{std}(T_{samp}(gt,ST),ST)$. Thus, $L_{samp}(gt,ST)$ denotes the total number of losses needed for an optimal completion of *gt*. Similarly, we can define $DL_{samp}(gt,ST)$ to be the total number of duplications and losses needed for a completion of *gt* that minimizes the duploss score.

The following theorem shows that the standard formula correctly computes the number of losses, when we treat incompleteness as due to taxon sampling

#### **Theorem 1**

*Given a binary rooted gene tree gt and a binary rooted species tree ST such that* $L(gt) \subseteq L(ST)$ *, the MRCA mapping defines a reconciliation that minimizes the number of duplications, the number of losses, and hence also the total number of duplications and losses, where we treat losses as due to sampling. Furthermore,* $L_{std}(gt,ST) = L_{samp}(gt,ST)$ *, which means the standard formula correctly computes the number of losses when we treat incompleteness as due to sampling.*

#### *Proof*

Consider *ST*(*gt*), the homeomorphic subtree of *ST* defined by the taxon set of *gt*. Since *gt* is complete with respect to *ST*(*gt*), the optimal reconciliation that minimizes duplications, losses, and their sum, is defined by $\mathcal {M}$, the MRCA mapping from *gt* to *ST*, and the standard formula correctly computes the number of losses for this reconciliation [18]. Note that for any completion *t* of *gt*, $L_{std}(t,ST) \ge L_{std}(gt,ST)$; in other words, the number of losses cannot decrease by making *gt* complete. Similarly, the number of duplications for *t* with respect to *ST* cannot be less than the number of duplications of *gt* with respect to *ST*. We will show that we can add all the remaining taxa into *gt* to produce a complete gene tree $t^*$ such that $L_{std}(t^*,ST)=L_{std}(gt,ST)$. Therefore, $t^*$ will be an optimal completion with respect to the $DL_{samp}$ problem. Furthermore, this will also imply that $L_{std}(gt,ST) = L_{samp}(gt,ST)$, as desired.

Recall the definition of the sets *UMMC* and *LMMC*, the upper and lower maximal missing clades, respectively. Since *gt* is not complete, there must be at least one missing taxon, and hence at least one maximal missing clade. If $\mathcal {M}(r(gt))=r(ST)$ then $UMMC=\emptyset$ and we set $gt'=gt$. Otherwise, $\mathcal {M}(r(gt)) \ne r(ST)$ and $UMMC \ne \emptyset$. Consider the path in *ST* from *r*(*ST*) down to $\mathcal {M}(r(gt))$, and the $m \ge 1$ subtrees that hang off that path before we reach $\mathcal {M}(r(gt))$. Note that each of these subtrees is an upper maximal missing clade. Let $gt'$ be the tree created by starting with *ST* and replacing the subtree of *ST* rooted at $\mathcal {M}(r(gt))$ by *gt*. Note also that the number of duplications has not changed, and that $L_{std}(gt',ST) = L_{std}(gt,ST)$.

If $LMMC = \emptyset$ we are done; otherwise, we now add the lower maximal missing clades to $gt'$ one at a time. Let $LMMC= \{t_1, t_2, \ldots , t_p\}$, so that $p \ge 1$. We will define a sequence of gene trees $gt_1, gt_2, \ldots , gt_p=t^*$, so that $gt_1$ is the result of adding clade $t_1$ to $gt'$, and $gt_i$ is the result of adding clade $t_i$ to $gt_{i-1}$ for $p \ge i \ge 2$. We will show that $L_{std}(gt_i,ST) = L_{std}(gt',ST)$ for $p \ge i \ge 1$, and that the number of duplications in $gt_i$ is the same as the number of duplications in $gt'$. Since $gt_p=t^*$ is a completion of *gt*, our theorem will be proven.

So consider $t=t_1$, the first lower maximal missing clade, and let *q* be the node in *ST* that is the parent of *r*(*t*) [i.e., $q = p(r(t))$]. Consider the edges (*x*, *y*) in $gt'$ with $y = p(x)$, such that *q* lies in the path between $\mathcal {M}(x)$ and $\mathcal {M}(y)$. Subdivide each such edge (creating a new node), and add *t* to $gt'$ by making its root the child of each such newly created node. Note that there must be at least one such edge in $gt'$ but there can be several such edges, and hence this step adds *t* at least once (and perhaps several times) to $gt'$. Note that when we add $t_1$ to $gt'$, we do not change the image under the MRCA mapping for any node *v* that is in $gt'$.

We now show that $t = t_1$ has the same number of duplications as *gt* with respect to *ST*. Clearly, any node in *t* is a speciation node (since *t* is a subtree of *ST*, which only has speciation nodes). Now consider a node *u* created by subdividing an edge (*x*, *y*), where *y* is the parent of *x* in $gt'$. One child of *u* is the root of *t* and the other child has an entirely disjoint leaf set; thus *u* is a speciation node. When we subdivide edge (*x*, *y*) we make *y* the parent of *u*. Therefore, $\mathcal {M}(u) \ne \mathcal {M}(y)$. Thus, *y* is a duplication node in $gt_1$ if and only if $\mathcal {M}(z)=\mathcal {M}(y)$ where *z* is the other child of *y* in $gt'$. But then *y* is a duplication node in $gt'$ if and only if *y* is a duplication node in $gt_1$, since the MRCA mapping does not change. Hence, no node in $gt'$ changes duplication/speciation status, and the newly added nodes are always speciation nodes. Therefore the number of duplication nodes does not change.

We now show that the number of losses does not change, i.e., $L_{std}(gt',ST)=L_{std}(gt_1,ST)$. Now consider an edge (*x*, *y*) that is subdivided through the addition of a node *u* that is made the parent of the subtree $t_1$. Then *x*, *y*,  and *u* all map (under $\mathcal {M}$) to different vertices in $ST(gt_1)$. Also, a simple case analysis (using the standard formula) verifies that $F(y,ST(gt')) = F(y,ST(gt_1)) + F(u,ST(gt_1))$. Since $F(z,ST(gt')) = F(z,ST(gt_1))$ for all other vertices $z \in V(gt')$, this means that the total number of losses does not change.

Therefore, the result of adding each lower maximal missing clade to $gt'$ does not imply any additional losses nor any additional duplications, and so also the total number of duplications and losses does not change. Let $t^* = t_p$ be the tree obtained after adding in all the missing maximal clades, and return $t^*$. The result then follows by induction on *p*. $\square$

### Incompleteness due to gene birth and death

As we will see, while the MRCA mapping is still an optimal reconciliation when gene trees are incomplete due to gene birth and death (implied from [18, 21]), the standard formula does not correctly compute the number of losses. We begin by summarizing some results that have already been established:

#### **Theorem 2**

(From [18, 21]:) *Given a binary rooted gene tree gt and a binary rooted species tree ST such that* $L(gt) \subseteq L(ST)$ *, the MRCA mapping defines a reconciliation that minimizes the number of duplications and the number of losses where we treat losses as due to gene birth and death. The set of speciation nodes in gt are those vertices* $v \in V_{int}(gt)$* that satisfy* $\mathcal {M}(v) \not \in \{\mathcal {M}(l), \mathcal {M}(r)\}$*, where l and r are the two children of v and* $\mathcal {M}$ *is the MRCA mapping from gt to ST; all other nodes are duplication nodes. Furthermore, we can compute the MRCA mapping, the set of duplication nodes, and the set of speciation nodes, in* $O(n+n')$ *time, where ST has n leaves and gt has* $n'$* leaves.*

#### *Proof*

Chauve et al. [18] proved that the MRCA mapping minimizes the losses required to reconcile *gt* with *ST*(*gt*) for complete gene trees, but the proof also applies to incomplete gene trees, treating incompleteness as due to gene birth and death. Górecki and Tiuryn [21] showed that the MRCA mapping minimizes the number of duplications required to reconcile *gt* with *ST*(*gt*), treating incompleteness as due to gene birth and death. Therefore, the MRCA mapping is optimal for all three scores (number of duplications, number of losses, and number of duplications plus losses), when treating incompleteness as due to gene birth and death.

It is easy to see that the duplication nodes in *gt* are those nodes that have $\mathcal {M}(v)=\mathcal {M}(l)$ or $\mathcal {M}(v)=\mathcal {M}(r)$ (where *l* and *r* are the two children of *v*, and $\mathcal {M}$ is the MRCA mapping), and all other nodes are speciation nodes. Since the MRCA mapping $\mathcal {M}$ can be computed in $O(n+n')$, where *ST* has *n* leaves and *gt* has $n'$ leaves, it follows that all these can be computed in $O(n+n')$ time. $\square$

However, the standard calculation for the number of losses can be incorrect when incompleteness is due to true biological loss! Consider the simple example $gt=((a,b),c)$ and $ST = ((a,(b,d)),c)$. Under the standard formula, $L_{std}(gt,ST)=0$, since $ST(gt)=gt$. Under the assumption that incompleteness is due to true biological loss, the genome for *d* does not have the gene. Because *d* is sister to *b* and all the other taxa have the gene, the gene must have been present in the parent of *d*, and lost on the branch leading to *d*. *Therefore, the standard formula for the number of losses can be incorrect when gene trees are incomplete due to gene birth and death (i.e., true biological loss)*.

## How to calculate losses

We now show how to calculate the number of losses for an incomplete gene tree *gt* and species tree *ST*, treating incomplete gene trees as due to gene birth and death. How this is defined will depend upon whether one assumes, *a priori*, that the gene is present in the genome of the common ancestor of the species in *ST* (i.e., at the root of *ST*). This needs to be taken into account as gene birth can happen only once whereas loss can happen repeatedly. Thus, this section shows how to calculate the following values:

- $L^*_{bd}(gt,ST)$, the minimum number of losses, under the assumption the gene is present in the common ancestor of the species in *ST* ($DL^*_{bd}(gt,ST)$ is defined similarly for the total number of duplications and losses), and
- $L_{bd}(gt,ST)$ the minimum number of losses *without* assuming the gene is present in the common ancestor of the species in *ST* ($DL_{bd}(gt,ST)$ is defined similarly for duplications and losses).

We now show how to compute the *number* of losses (i.e., $L_{bd}(gt,ST)$ and $L^*_{bd}(gt,ST)$), using the fact that the MRCA mapping defines an optimal reconciliation.

### **Theorem 3**

*Let gt be a gene tree and ST a species tree such that* $L(gt) \subseteq L(ST)$ *. Then,* $L_{bd}(gt,ST) = \sum _{u \in V_{int}(gt)}F(u,ST)$ *, and* $L^*_{bd}(gt,ST) = L_{bd}(gt,ST) + |UMMC(gt,ST)|.$ *Furthermore, these values can be calculated in* $O(n+n')$ *time, where ST has n leaves and gt has* $n'$ *leaves.*

### *Proof*

Note that we use a modification of the standard formula, *F*(*u*, *ST*), so that we do not replace *ST* by *ST*(*gt*) as was done in [18, 19].

**Derivation of** $L_{bd}(\mathbf{gt,ST})$ Recall that $L_{bd}(gt,ST)$ does not assume that the most recent common ancestor of the species in *ST* has the gene. Since gene birth can happen only once (although loss can happen repeatedly), we begin by determining the location of the gene birth. If $\mathcal {M}(r(gt))=r(ST)$, then the gene is born before *r*(*ST*), and is present at the root of *ST*. Otherwise, it is easy to see that the location of the gene birth that minimizes the number of losses is the edge above $\mathcal {M}(r(gt))$. Now consider the modification of the standard formula (i.e., using *ST* instead of *ST*(*gt*)):

2 $$\begin{aligned} L_{bd}(gt,ST) = \sum _{u \in V_{int}(gt)} F(u,ST). \end{aligned}$$

It is easy to see that this correctly returns the number of inserted subtrees, and hence the number of losses.

**Fig. 1:**
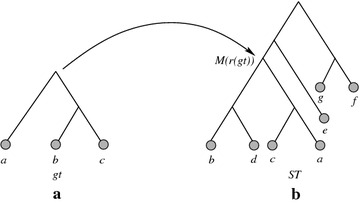
An example showing the difference between $L_{bd}(gt,ST)$ and $L^*_{bd}(gt,ST)$. **a** A gene tree $gt = ((b,c),a)$, **b** a species tree $ST = ((((a,c),(b,d)),e),(f,g))$. Here, $\mathcal {M}(r(gt)) \ne r(ST)$, and $UMMC(gt,ST) = \{\{e\},\{f,g\}\}$. $L_{bd}(gt,ST)$ is the number of losses required to reconcile *gt* with *ST* according to Eq. 2, and we get $L^*_{bd}(gt,ST)$ by adding |*UMMC*(*gt*, *ST*)| to $L_{bd}(gt,ST)$

**Derivation of **$L^*_{bd}(\mathbf{gt,ST})$ By definition of $L^*_{bd}(gt,ST)$, the gene is assumed to be present at the root of the species tree *ST*. If $\mathcal {M}(r(gt))=r(ST)$, then $UMMC(gt,ST) = \emptyset$, and the result follows. However, if $\mathcal {M}(r(gt)) \ne r(ST)$, the gene must be present on the path between *r*(*ST*) and $\mathcal {M}(r(gt))$. Since the gene is not present in any leaf that is not below $\mathcal {M}(r(gt))$, to minimize losses, the gene must be lost on every edge off that path, since such edges lead to subtrees that do not have the gene present in any leaf. Note that if $\mathcal {M}(r(gt)) \ne r(ST)$, then the number of edges that lead off that path is $|UMMC(gt,ST)| = d(\mathcal {M}(r(gt)),r(ST))+1$. Since the gene must be lost on each of those edges, and the total number of losses is the sum of this value and the number of losses that occur within the subtree rooted at $\mathcal {M}(r(gt))$, it follows that $L^*_{bd}(gt,ST) = L_{bd}(gt,ST) + |UMMC(gt,ST)|$. Figure 1 illustrates an example distinguishing $L_{bd}(gt,ST)$ and $L^*_{bd}(gt,ST)$. The running time follows easily from the fact that the MRCA mapping can be computed in linear time [22]. $\square$

Now one of the most important questions in terms of estimating the optimal species tree is – given a set $\mathcal {G}$ of (possibly incomplete) gene trees, is the species tree that minimizes $\sum _{gt\in \mathcal {G}}L^*_{bd}(gt,ST)$ or $\sum _{gt\in \mathcal {G}}L_{bd}(gt,ST)$ different than the one that minimizes $\sum _{gt\in \mathcal {G}}L_{std}(gt,ST)$? If the same species tree optimizes both ways of calculating losses, then defining loss differently is not that important in the context of phylogenomic analyses. But this is not necessarily true, as we will show in the following theorem.

### **Theorem 4**

*Let* $\mathcal {G}$ *be a set of incomplete gene trees and* $ST_{bd},$ $ST^*_{bd}$ *and* $ST_{std}$ *are the species trees that minimizes* $\sum _{gt\in \mathcal {G}}L_{bd}(gt,ST),$ $\sum _{gt\in \mathcal {G}}L^*_{bd}(gt,ST)$ *and* $\sum _{gt\in \mathcal {G}}L_{std}(gt,ST)$ *, respectively. Then* $ST_{std}$ *is not necessarily identical to* $ST_{bd}$ *or* $ST^*_{bd}.$

### *Proof*

**Fig. 2:**
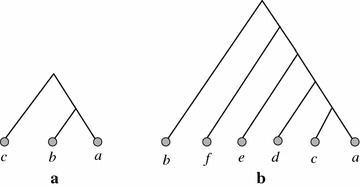
Gene tree topologies used in Theorem 4 to prove that the species tree which is optimal under the $L_{std}$ criterion is not necessarily optimal under the $L_{bd}$ criterion. **a** Gene tree topology $tp_1$ and **b** gene tree topology $tp_2$

We will present an input of 14 gene trees and a species tree $ST_1$ that optimizes the $L_{std}$ criterion and that provably is not optimal for the $L_{bd}$ criterion. Consider the two gene tree topologies $tp_1 = ((a,b),c)$ and $tp_2 = (b,(f,(e,(d,(c,a)))))$ as shown in Fig. 2a, b. Let $\mathcal {G}$ be a set of 14 gene trees, with eight gene trees having topology $tp_1$ and the remaining six gene trees having topology $tp_2$. It is easy to verify that the species tree $ST_1$ with topology $tp_2$ minimizes $\sum _{gt\in \mathcal {G}}L_{std}(gt,ST_1)$. Here, $\sum _{gt\in \mathcal {G}}L_{std}(gt,ST_1) = 8*3 + 6*0 = 24$. Any other species tree will result into more than 24 losses, and the reason is as follows. There are three tree topologies on leafset $\{a,b,c\}$: ((*a*, *b*), *c*), ((*a*, *c*), *b*) and ((*b*, *c*), *a*). Reconciling $tp_1$ with ((*a*, *c*), *b*) or ((*b*, *c*), *a*) requires 3 losses. Therefore, any species tree *T* such that $T(tp_1)$ is not identical to $tp_1 = ((a,b),c)$ requires $8*3 = 24$ losses to reconcile the eight gene trees having topology $tp_1$ with *T*. Therefore, to achieve less than 24 losses, $T(tp_1)$ should be identical to $tp_1$. We now calculate the number of losses required to reconcile $tp_2$ with a species tree *T* such that $T(tp_1) = ((a,b),c)$. Note that, $tp_2(tp_1) = ((a,c),b).$ Reconciling ((*a*, *c*), *b*) with ((*a*, *b*), *c*) requires three losses. Then taking $\{d,e,f\}$ into consideration, it is quite easy to verify that it requires more than three losses to reconcile $tp_2$ with a species tree *T* such that $T(tp_1) = ((a,b),c)$. Hence, there is no species tree *T* so that $\sum _{gt\in \mathcal {G}}L_{std}(gt,T) < 24$. Therefore, $ST_1 = tp_2$ minimizes $\sum _{gt\in \mathcal {G}}L_{std}(gt,ST_1)$.

However, the species tree $ST_2 = (((((a,b),c),d),e),f)$ minimizes $\sum _{gt\in \mathcal {G}}L_{bd}(gt,ST_2)$. Here $\sum _{gt\in \mathcal {G}}L_{bd}(gt,ST_2) = 8*3 + 6*6 = 60$, which is less than $\sum _{gt\in \mathcal {G}}L_{bd}(gt,ST_1=tp_2) = 8*9 + 6*0 = 72$.

Therefore, $ST_{std}$ is not necessarily same as $ST_{bd}$. Then the fact that $ST_{std}$ is not necessarily identical to $ST^*_{bd}$ immediately follows. $\square$

## Algorithms to find species trees

Here we address the problem of finding a species tree that has a minimum total number of duplications and losses, treating incompleteness as due to true biological loss. Prior results on GTP include a branch-and-bound algorithm in [23] based on techniques from [18], a randomized hill-climbing heuristic presented in [4], a probabilistic and computationally expensive method for co-estimating gene and species trees [1], and dynamic programming based solutions by Hallett and Lagergren [15], Bayzid et al. [20] and Chang et al. [24]. However, none of these studies takes the reasons of incompleteness into account, and we have already shown that the standard calculation for losses can be incorrect when incompleteness is due to true biological loss.

In this section, we derive a different approach for the GTP problems, treating incomplete gene trees as due to true biological loss (i.e., minimizing $L_{bd}(gt,ST)$ or $L^*_{bd}(gt,ST)$). The techniques we propose can be used to solve GTP exactly for small datasets, or approximately (though without any guaranteed error bounds) on larger datasets. The approach we take here is based on [20] (see also [15, 25, 26], which use very similar techniques). Bayzid et al. [20] provided a graph-theoretic formulation for $MGDL_{std}$, whereby an optimal solution to $MGDL_{std}$ corresponded to finding a minimum weight maximum clique inside a graph called the “Compatibility Graph”. The nodes of the compatibility graph correspond to “subtree-bipartitions”, a concept Bayzid et al. [20] introduced and we will also use. Bayzid et al. [20] showed how to find a minimum weight max clique using a dynamic programming approach. We will use the same graph-theoretic formulation as in [20], but modify the weights appropriately, to show that the optimal solution to $MGDL^*_{bd}$ still corresponds to a minimum weight max clique. The DP algorithm in [20] can then be used directly to find the optimal solution to $MGDL^*_{bd}$. To achieve this, we first derive an efficient formula for $L_{bd}(gt,ST)$ (and $L^*_{bd}(gt,ST)$, similar to the one derived in [17] for $L_{std}(gt,ST)$, but somewhat more involved.

We will let $\mathcal {D}_{gt,ST}$ denote the set of duplication nodes in *gt* with respect to *ST* and $\mathcal {S}_{gt,ST}$ denote the set of speciation nodes in *gt* with respect to *ST*. When *gt* and *ST* are known, we may write these as $\mathcal {D}$ and $\mathcal {S}$. The calculation for the number of losses depends on how we interpret incompleteness in gene trees. Therefore, rather than having a single optimization problem like *MGDL*, we have variants of this problem depending on how we treat incompleteness. As shown in Theorem 1, the term *MGDL* in the literature refers to $MGDL_{std}$, which (by Theorem 1) is identical to $MGDL_{samp}$. Here, we consider the optimization problems $MGDL^*_{bd}$, where we treat incompleteness as due to gene birth and death. And therefore, we also consider $MGDL_{bd}$, $MGL^*_{bd}$, and $MGL_{bd}$.

### Basic material

#### Subtree-bipartitions

Let *T* be a rooted binary tree and *u* an internal node in *T*. The *subtree-bipartition* of *u*, denoted by $\mathcal {SBP}_T(u)$, is the unordered pair $(c_T(l)|c_T(r))$, where *l* and *r* are the two children of *u*. Note that subtree-bipartitions are not defined for leaf nodes. The set of subtree-bipartitions of a tree *T* is denoted by $\mathcal {SBP}_T = \{\mathcal {SBP}_{T}(u): u \in V_{int}(T)\}$. Furthermore, any pair *A* and *B* of disjoint subsets of $\mathcal {X}$ also define a subtree-bipartition (though we may refer to these as *candidate* subtree-bipartitions to emphasize this).

*Subtree-bipartition domination* Let $BP_i = (P_{i_1}|P_{i_2})$ and $BP_j = (P_{j_1}|P_{j_2})$ be two subtree-bipartitions. We say that $BP_i$ is *dominated by* $BP_j$ (and conversely that $BP_j$ *dominates* $BP_i$) if either of the following two conditions holds: (1) $P_{i_1} \subseteq P_{j_1}$ and $P_{i_2} \subseteq P_{j_2},$ or (2) $P_{i_1} \subseteq P_{j_2}$ and $P_{i_2} \subseteq P_{j_1}.$ We say that subtree-bipartition (*A*|*B*) is dominated by a species tree *T* if one of *T*’s subtree-bipartitions dominates (*A*|*B*). Bayzid *et al.* showed that an internal node *u* in a gene tree *gt* is a duplication node with respect to a species tree *ST* if $\mathcal {SBP}_{gt}(u)$ is dominated by *ST* [20]. Finally, for a set $\mathcal {G}$ of gene trees on taxon set $\mathcal {X}$ and for any candidate subtree-bipartition (*A*|*B*), we let $W_{dom}(A|B)$ be the total number of subtree-bipartitions in $\mathcal {G}$ that are dominated by (*A*|*B*).

*Subtree-bipartition containment and compatibility* We say that $BP_i$ *contains* $BP_j$ if $P_{j_1} \cup P_{j_2} \subseteq P_{i_1}$ or $P_{j_1} \cup P_{j_2} \subseteq P_{i_2}$, and that $BP_i$ and $BP_j$ are *disjoint* if $[P_{i_1} \cup P_{i_2}] \cap [P_{j_1} \cup P_{j_2}] = \emptyset$. We say that two subtree bipartitions are *compatible* if they are disjoint or one contains the other.

*The compatibility graph *$CG(\mathcal {G})$ Let $\mathcal {G}$ be a set of rooted binary gene trees on the set $\mathcal {X}$ of *n* taxa. The *compatibility graph* $CG(\mathcal {G})$ has a vertex for a subtree-bipartition defined on $\mathcal {X}$, and there is an edge between two vertices if and only if the associated subtree-bipartitions are compatible.

#### Deep coalescence and the MDC problem 

*Deep coalescence* (also called *incomplete lineage sorting*, or ILS) refers to the failure of alleles to coalesce (looking backwards in time) into a common ancestral allele until deeper than the most recent speciation events [27]. One of the measures for incongruence between a gene tree and a species tree under ILS is *XL*(*gt*, *ST*), the number of extra lineages defined for the pair *ST* and *gt* [27]. For a gene tree *gt* and a species tree *ST* such that $L(gt) \subseteq L(ST),$ the number of extra lineages (summing over all edges) is defined to be

$$\begin{aligned} XL(gt,ST) = \displaystyle \sum _{e' \in E(ST^*(gt))} XL(gt,e'), \end{aligned}$$

where $XL(gt,e')$ is the number of extra lineages on $e'$.

Minimize deep coalescence (MDC) is an optimization problem for estimating species trees in the presence of ILS. MDC problem can be defined as follows.

- **Problem**          **Minimize deep coalescence (MDC)**
- INPUT             A set $\mathcal {G}$ of gene trees.
- OUTPUT          A species tree *ST* such that $\sum _{gt \in \mathcal {G}} XL(gt,ST)$ is minimized.

This problem is also NP-hard [17], and software for the problem exists in Phylonet [28] and iGTP [5], among others. We now describe theoretical material leading to the algorithmic approach in Phylonet [26].

##### **Definition 5**

(*From* [26]) For $B \subseteq \mathcal {X}$ and gene tree *gt*, we set $k_B(gt)$ to be the number of B-maximal clusters in *gt*, where a *B-maximal cluster* is a cluster $Y \subseteq L(gt)$ such that $Y \subseteq B$ but no other cluster of *gt* containing *Y* is a subset of *B*.

##### **Definition 6**

We define $W_{xl}(x,gt)$ for *x* either a subtree-bipartition or a subset of $\mathcal {X}$, as follows. If $x \subseteq \mathcal {X}$, then we set $W_{xl}(x,gt) = 0$ if $x \cap L(gt) = \emptyset$ and otherwise $W_{xl}(x,gt)=k_x(gt)-1$. If *x* is a subtree-bipartition, then we let $B = p \cup q$ for $x=(p|q)$, and we set $W_{xl}(x,gt) = 0$ if $B \cap L(gt) = \emptyset$, and otherwise $W_{xl}(x,gt) = k_B(gt) -1$. For a set $\mathcal {G}$ of gene trees and *ST* a species tree, we set $W_0 = \sum _{gt \in \mathcal {G}}\sum _{x \in \mathcal {X}}W_{xl}(\{x\},gt)$.

Yu et al. [26] showed that for any edge *e* in *ST*, where *B* is the cluster below *e*, then $k_B(gt)$ is the number of lineages going through edge *e*, and so $k_B(gt)-1$ is the number of extra lineages going through *e*. They defined weights on potential species tree clusters *B* by $W_{mdc}(B,gt)$ = 0 if $B \cap L(gt) = \emptyset$ and otherwise $W_{mdc}(B,gt) = k_B(gt)-1$ (i.e., $W_{mdc}$ is defined for clusters while $W_{xl}$ is defined for subtree-bipartitions), and extended this to a set $\mathcal {G}$ of gene trees by $W'_{mdc}(B) = \sum _{gt \in \mathcal {G}} W_{mdc}(B,gt)$, and then to a set *C* of clusters by $W''_{mdc}(C)=\sum _{B \in C} W'_{mdc}(B)$. From this, it follows easily that a set *C* of $n-1$ compatible clusters minimizing $W''_{mdc}(C)$ defines a rooted binary species tree with a minimum MDC score.

### Deriving $L_{bd}(gt,ST)$ and $L^*_{bd}(gt,ST)$

#### **Theorem 7**

[From [17]] *Let gt be a rooted binary gene tree, ST a rooted binary species tree and *$\mathcal {D}$ *the set of duplication nodes in gt with respect to ST. Then*

$$\begin{aligned} L_{std}(gt,ST) = XL(gt,ST(gt)) + 2|\mathcal {D}| + |V(gt)| - |V(ST(gt))|. \end{aligned}$$

We now derive formulas for $L_{bd}(gt,ST)$ and $L^*_{bd}(gt,ST)$; to obtain formulas for $DL_{bd}(gt,ST)$ and $DL^*_{bd}(gt,ST)$, simply add $|\mathcal {D}_{gt,ST}|$. Recall that in the definition of *F*(*u*, *T*) given in Eq. 1, losses are associated with internal nodes, and the total number of losses is defined as the sum of losses associated to each internal node. However, the definition of the number of losses corresponding to a node can be rewritten in terms of edges, as we now show. Let $D(s,s')$ be the number of edges in the path in *ST* between *s* and $s'$. Therefore, $D(s,s')$ can be defined as follows.

$$\begin{aligned} D(s,s') = \left\{ \begin{array}{ll} d(s,s') + 1 &{} \quad \text {if}\, d(s,s') \ge 1, \\ d(s,s') &{} \quad \text {if}\, d(s,s') = 0.\\ \end{array} \right. \end{aligned}$$

Then, for a vertex *u* in *gt* with children *r* and *l*, we can rewrite Eq. 1 as follows:

$$\begin{aligned} F(u,ST) = \left\{ \begin{array}{l} D(\mathcal {M}(r), \mathcal {M}(u)) + D(\mathcal {M}(l),\mathcal {M}(u)),\\ \text {if}\mathcal {M}(r) \ne \mathcal {M}(u) = \mathcal {M}(l).\\ D(\mathcal {M}(r), \mathcal {M}(u)) + D(\mathcal {M}(l),\mathcal {M}(u)),\\ \text {if}\mathcal {M}(l) \ne \mathcal {M}(u) = \mathcal {M}(r).\\ (D(\mathcal {M}(r), \mathcal {M}(u)) - 1) + (D(\mathcal {M}(l), \mathcal {M}(u)) - 1),\\ \text {if}\mathcal {M}(u) \not \in \{\mathcal {M}(l), \mathcal {M}(r)\}.\\ D(\mathcal {M}(r), \mathcal {M}(u)) + D(\mathcal {M}(l), \mathcal {M}(u)),\\ \text {if}\mathcal {M}(r) = \mathcal {M}(u) = \mathcal {M}(l).\\ \end{array} \right. \end{aligned}$$

It is easy to see that in all three branches of the equation above, the two terms of the sum correspond to the edges connecting *u* to its two children *l* and *r*. (The second term in the first branch and both terms in the third branch are 0, but we wrote them in terms of the function *D*(., .) for convenience.) Let *p*(*x*) be the parent of *x* in a tree *T*. Therefore, we can associate gene losses to edges $e = (x,p(x))$ instead of nodes, as follows:

$$\begin{aligned} \mathcal {M}D(e) = D(\mathcal {M}(x),\mathcal {M}(p(x)), \text {and} \end{aligned}$$

$$\begin{aligned} edgeloss_{ST}(e) = \left\{ \begin{array}{ll} \mathcal {M}D(e) &{} \quad \text {if}\, p(x) \in \mathcal {D}_{gt,ST},\\ \mathcal {M}D(e) - 1 &{} \quad \text {otherwise.}\\ \end{array} \right. \end{aligned}$$

We use the subscript *ST* in $edgeloss_{ST}(e)$ to emphasize the fact that the distance is taken within the tree *ST* and not within *ST*(*gt*). Therefore,

$$\begin{aligned} \sum _{u \in V_{int}(gt)} F(u,ST) = \sum _{e \in E(gt)} edgeloss_{ST}(e). \end{aligned}$$

#### **Lemma 8**

*For all gene trees gt and species trees ST with* $L(gt) \subseteq L(ST),$

3 $$\begin{aligned} L_{bd}(gt,ST) = \displaystyle \sum _{e \in E(gt)} \mathcal {M}D(e) - |E(gt)| + 2 |\mathcal {D}|, \end{aligned}$$

*and for a set* $\mathcal {G}$ *of gene trees,*

4 $$\begin{aligned} L_{bd}(\mathcal {G},ST)= &{} \displaystyle \sum _{gt \in \mathcal {G}}L_{bd}(gt,ST) \\= &{} \displaystyle \sum _{gt \in \mathcal {G}} \displaystyle \sum _{e \in E(gt)} \mathcal {M}D(e) \\ \quad &- \displaystyle \sum _{gt \in \mathcal {G}}|E(gt)| + 2 \displaystyle \sum _{gt \in \mathcal {G}} |\mathcal {D}_{gt,ST}|. \end{aligned}$$

*Finally, equalities concerning* $DL_{bd}(gt,ST)$* and* $DL_{bd}(\mathcal {G},ST)$* can be obtained from these equalities by adding* $|\mathcal {D}_{gt,ST}|$* and* $|\mathcal {D}_{\mathcal {G},ST}|$*, where* $|\mathcal {D}_{\mathcal {G},ST}| = \sum _{gt \in \mathcal {G}} |\mathcal {D}_{gt,ST}|$.

#### *Proof*

We partition all the non-root nodes in *gt* into two sets: *CD* (children of duplications), consisting of those nodes whose parents are duplication nodes, and *CS* (children of speciations), consisting of those nodes whose parents are speciation nodes. Note that every edge $(x,p(x)) \in E(gt)$ can be associated with the set containing *x*. Therefore,

5 $$\begin{aligned} L_{bd}(gt,ST)= &{} \displaystyle \sum _{e \in E(gt)} edgeloss_{ST}(e)\nonumber \\= & {} \displaystyle \sum _{{x \in CD}} \mathcal {M}D(x,p(x)) \\ \quad & + \displaystyle \sum _{{x \in CS}} (\mathcal {M}D(x,p(x)) - 1) \nonumber \\= & {} \displaystyle \sum _{e \in E(gt)} \mathcal {M}D(e) - |CS|. \end{aligned}$$

Since each internal node has two children, clearly the number of vertices *x* for which *p*(*x*) is a speciation node is twice the number $|\mathcal {S}|$ of speciation nodes; therefore $L_{bd}(gt,ST) = \displaystyle \sum\nolimits_{e \in E(gt)} \mathcal {M}D(e) - 2 |\mathcal {S}|.$ Since each internal node is a speciation node or a duplication node, it follows that $2(|\mathcal {D}| + |\mathcal {S}|) = |E(gt)|$, and the result follows. $\square$

Let *L*(*gt*, *e*) be the number of lineages that go through edge $e \in E(ST)$; thus, $XL(gt,e) = L(gt,e) - 1$, and so

6 $$\begin{aligned} XL(gt,ST) = \displaystyle \sum _{e' \in E(ST^*(gt))} L(gt,e') - |E(ST^*(gt))|. \end{aligned}$$

#### **Lemma 9**

*For any gene tree gt and species tree ST, *$\sum _{e \in E(gt)} \mathcal {M}D(e) = \sum _{e' \in E(ST^*(gt))} L(gt,e'),$* and (by Eq.* 6*)*

7 $$\begin{aligned} XL(gt,ST) = \displaystyle \sum _{e \in E(gt)} \mathcal {M}D(e) - |E(ST^*(gt))|. \end{aligned}$$

Thus, for a set $\mathcal {G}$ of gene trees and species tree *ST*,

$$\begin{aligned} XL(\mathcal {G},ST)= & {} \displaystyle \sum _{gt \in \mathcal {G}} XL(gt,ST) = \displaystyle \sum _{gt \in \mathcal {G}} \displaystyle \sum _{e \in E(gt)} \mathcal {M}D(e) -\sum _{gt \in \mathcal {G}} |E(ST^*(gt))|. \end{aligned}$$

#### *Proof*

We establish the first equality, since the remaining ones follow directly from it. Consider the lists of edges in paths in *ST* from $\mathcal {M}(x)$ to $\mathcal {M}(p(x))$, as *x* ranges over the internal vertices in *gt*. It is easy to see that the number of occurrences of an edge $e' \in E(ST^*(gt))$ in these lists is $L(gt, e')$ (the number of lineages through $e'$). Also, the edges $e \in E(ST) - E(ST^*(gt))$ will not be present in these lists, since these are the edges incident on the missing clades in *ST* with respect to *gt*. Therefore, the sum of the lengths of these lists is equal to $\sum _{e \in E(gt)} \mathcal {M}D(e)$ and also equal to $\sum _{e \in ST^*(gt)}L(gt,e)$. $\square$

#### **Theorem 10**

*For all gene trees gt, sets* $\mathcal {G}$ *of gene trees, and species trees ST,* $L_{bd}(gt,ST) = XL(gt,ST) + 2 |\mathcal {D}| + |E(ST^*(gt))| - |E(gt)|$ *, and*

8 $$\begin{aligned} L_{bd}(\mathcal {G},ST) = XL(\mathcal {G},ST) + 2 \displaystyle \sum _{gt \in \mathcal {G}} |\mathcal {D}_{gt,ST}| + \sum _{gt\in \mathcal {G}}(|E(ST^*(gt))| - |E(gt)|). \end{aligned}$$

#### *Proof*

Follows from Lemmas 8 and 9. $\square$

#### **Corollary 1**

*For all gene trees gt and species trees ST,*

$$\begin{aligned} L^*_{bd}(gt,ST)= \,& {} L_{bd}(gt,ST) + |UMMC(gt,ST)|\\= \,& {} XL(gt,ST) + 2 |\mathcal {D}_{gt,ST}| +\,|E(ST^*(gt))| - |E(gt)|\\&+\,|UMMC(gt,ST)|.\\ DL^*_{bd}(gt,ST)= \,& {} L_{bd}(gt,ST) + |UMMC(gt,ST)| +\,|\mathcal {D}_{gt,ST}|\\= \,& {} XL(gt,ST) + 3 |\mathcal {D}_{gt,ST}| +\,|E(ST^*(gt))| - |E(gt)|\\&+\,|UMMC(gt,ST)| \end{aligned}$$

#### *Proof*

The equalities concerning $L^*_{bd}$ follow from Theorems 3 and  10. The equalities concerning $DL^*_{bd}$ follow by adding $|\mathcal {D}_{gt,ST}|$. $\square$

### Assigning weights to subtree-bipartitions

To use the graph-theoretic formulation of $MGDL^*_{bd}$, we have to assign weights to each node in the compatibility graph, $CG(\mathcal {G})$, where $\mathcal {G}$ is the input set of gene trees, so that a minimum weight clique of $n-1$ vertices defines an optimal solution to $MGDL^*_{bd}(\mathcal {G})$. We will define weights $W_{xl}(v), W_{dom}(v), W_{EC}(v)$, and $W_{MMC}(v)$ to each subtree-bipartition (i.e., node in the compatibility graph), and set

$$\begin{aligned} W_{MGDL^*_{bd}}(v) = W_{xl}(v) -3W_{dom}(v) +W_{EC}(v) +W_{MMC}(v). \end{aligned}$$

We then prove (see Theorem 11) that a set of $n-1$ compatible subtree-bipartitions that has minimum total weight defines a species tree that optimizes $MGDL^*_{bd}$. Note that weights $W_{xl}(v)$ and $W_{dom}(v)$ have already been defined. Hence, all that remains is to define $W_{EC}(v)$ and $W_{MMC}(v)$, and then to prove Theorem 11.

*Calculating* $W_{EC}(v)$ *and* $|E(ST^*(gt))|$: We now show how to define weight $W_{EC}(v,gt)$ for every vertex *v* in the compatibility graph $CG(\mathcal {G})$ so that for all species trees *ST*, $|E(ST^*(gt))|$ is the sum of the vertex weights for the $n-1$ clique ${\mathcal {C}}$ in $CG(\mathcal {G})$ corresponding to *ST*. To count the number of edges in $E(ST^*(gt))$, we need to exclude those edges from *E*(*ST*) that are incident on a clade that is missing in *gt*. For a vertex *v* associated with the subtree-bipartition (*p*|*q*), we define $W_{EC}(v,gt)$ as follows (swapping *p* and *q* as needed):

9 $$\begin{aligned} W_{EC}(v,gt) = \left\{ \begin{array}{ll} 0 &{} \quad \text {if}\;p \cap L(gt) = \emptyset \,\, \text {and} \,\, q \cap L(gt) \in \{L(gt),\emptyset \}\\ 1 &{} \quad \text {if}\; p \cap L(gt) = \emptyset \,\, \text {and} \,\, \emptyset \ne q \cap L(gt)\subsetneq L(gt)\\ 2 &{} \quad \text {otherwise.} \end{array} \right. \end{aligned}$$

Then, $|E(ST^*(gt))| = \sum _{u\in \mathcal {SBP}_{ST}} W_{EC}(u,gt)$. We set $W_{EC}(v) = \sum _{gt \in \mathcal {G}} W_{EC}(v,gt)$. Then, for any species tree *ST* and set $\mathcal {G}$ of gene trees,

10 $$\begin{aligned} \sum _{gt \in \mathcal {G}}|E(ST^*(gt))| = \sum _{v\in {\mathcal {C}}} W_{EC}(v), \end{aligned}$$

where ${\mathcal {C}}$ is the clique in $CG(\mathcal {G})$ that corresponds to *ST*.

*Calculating* $W_{MMC}(v)$ *and* |*UMMC*(*gt*, *ST*)| We now show how to assign the weight $W_{MMC}(v,gt)$ to each vertex *v* of the compatibility graph so that for all species trees *ST*, |*UMMC*(*gt*, *ST*)| is the sum of the weights over all the vertices of the clique ${\mathcal {C}}$ in $CG(\mathcal {G})$ corresponding to *ST*. Recall that *UMMC*(*gt*, *ST*) is the set of upper maximal missing clades in *ST*. For a vertex *v* associated with the subtree-bipartition (*p*|*q*), we define $W_{MMC}(v,gt)$ as follows (swapping *p* and *q* as needed):

11 $$\begin{aligned} W_{MMC}(v,gt) = \left\{ \begin{array}{ll} 1 &{} \quad \text {if}\,\, p \cap L(gt) = \emptyset \,\, \text {and} \, q \cap L(gt) = L(gt) (or\,vice-versa)\\ 0 &{} \quad \text {otherwise.} \end{array} \right. \end{aligned}$$

Then $|UMMC(gt,ST)| = \sum _{u\in \mathcal {SBP}_{ST}} W_{MMC}(u,gt).$ Finally, we set $W_{MMC}(v) = \sum _{gt \in \mathcal {G}} W_{MMC}(v,gt).$ Then, for any species tree *ST* and set $\mathcal {G}$ of gene trees,

12 $$\begin{aligned} \sum _{gt \in \mathcal {G}} |UMMC(gt,ST)| = \sum _{v\in {\mathcal {C}}} W_{MMC}(v), \end{aligned}$$

where ${\mathcal {C}}$ is the clique in $CG(\mathcal {G})$ that corresponds to *ST*.

We can extend the $MGDL^*_{bd}$ techniques to allow for losses and duplications to have different costs, as follows. Let $c_d$ be the cost of a duplication and assume the cost of a loss ($c_l$) is 1. (Note that, our techniques work for any arbitrary $c_d$ and $c_l$.) Let $|\mathcal {D}_{\mathcal {G},ST}| = \sum _i^k |\mathcal {D}_{gt_i,ST}|$, and set $DL^*_{bd}(\mathcal {G},ST,c_d) = c_d*|\mathcal {D}_{\mathcal {G},ST}| + L^*_{bd}(\mathcal {G},ST).$ Let $MGDL^*_{bd}(\mathcal {G},c_d)$ be the problem that takes a set $\mathcal {G}$ of gene trees and duplication cost $c_d$ as input, and finds the species tree that minimizes the weighted duploss score $DL^*_{bd}(\mathcal {G},ST,c_d)$. Let $W^{c_d}_{MGDL^*_{bd}}(v) = W_{xl}(v)-(c_d+2)W_{dom}(v) + W_{EC}(v) + W_{MMC}(v)$. If $c_d=1$, we omit the superscript $c_d$ and write $W_{MGDL^*_{bd}}(v)$.

#### **Theorem 11**

*Let* $\mathcal {G}=\{gt_1, gt_2, \ldots , gt_k\}$ *be a set of binary rooted gene trees on set* $\mathcal {X}$ *of n species, and set the weights on the vertices in the compatibility graph using* $W^{c_d}_{MGDL^*_{bd}}(v).$ *(a) A set of subtree-bipartitions in an* $(n-1)$ *-clique of minimum weight in* $CG(\mathcal {G})$ *defines a binary species tree ST that minimizes* $DL^*_{bd}(\mathcal {G}, ST,c_d)$ *. Furthermore, the weighted duploss score of ST is given by* $W_0 + W^{c_d}_{MGDL^*_{bd}}({\mathcal {C}}) + c_d(N -k)$ *, where* $N = \sum _{i=1}^k n_i.$ *(b) If we reset the weights to be* $W_{MGL^*_{bd}}(v) = W_{MGDL^*_{bd}}(v) +W_{dom}(v)$ *, then a set of subtree-bipartitions in an* $(n-1)$ *-clique of minimum weight in* $CG(\mathcal {G})$ *defines a binary species tree ST that minimizes* $L^*_{bd}(\mathcal {G},ST).$

#### *Proof*

We prove (a), since (b) follows directly from (a). Let ${\mathcal {C}}$ be a clique of size $n-1$ in $CG(\mathcal {G})$ and *ST* the associated species tree. Let $\mathcal {SBP}_{dom}(gt,ST)$ be the set of subtree-bipartitions in *gt* that are dominated by a subtree-bipartition in *ST*. Note that $|\mathcal {SBP}_{dom}(gt,ST)|$ is the number of speciation nodes in *gt* with respect to *ST* [20]. Therefore, the total number of speciation nodes in $\mathcal {G}$ is $\sum _{i=1}^k |\mathcal {SBP}_{dom}(gt_i,ST)| =\sum _{v \in V_{int}(ST)}W_{dom}(v)$. Also, $\sum _{v \in {\mathcal {C}}} W_{xl}(v) = \sum _{i=1}^k XL(gt_i,ST)$, and $\sum _{i=1}^k |\mathcal {D}_{gt_i,ST}|=\sum _{i=1}^k (n_i-1) -\sum _{v \in {\mathcal {C}}}W_{dom}(v)$, where $n_i$ is the number of leaves in $gt_i$. Finally, since all gene trees are rooted binary trees, $|E(gt_i)|=2n_i-2$ and $|V_{int}(gt_i)|=n_i-1$. Recall that $W_0$ is the number of extra lineages contributed by the leaf set of the species tree (Definition 6). Therefore,

$$\begin{aligned} DL^*_{bd}(\mathcal {G}, ST,c_d)= & {} \sum _{i=1}^k (c_d*|\mathcal {D}_{gt_i,ST}| + L^*_{bd}(gt_i, ST))\\= & {} \sum _{i=1}^k[ XL(gt_i, ST) + (c_d+2)|\mathcal {D}_{gt_i,ST}| +~ |UMMC(gt_i,ST)|\\&+ ~|E(ST^*(gt_i))| - |E(gt_i)|] \text{(by } \text{ Cor. } \text{1) }\\=\, & {} W_0 + \sum _{v \in {\mathcal {C}}}W_{xl}(v) + \sum _{i=1}^k(c_d+2)(n_i-1)- (c_d+2) \sum _{v \in {\mathcal {C}}}W_{dom}(v)\\&+ \sum _{v \in {\mathcal {C}}}W_{MMC}(v) ~+ \sum _{v \in {\mathcal {C}}}W_{EC}(v) - \sum _{i=1}^k (2n_i - 2) \end{aligned}$$

(by Eqs. 10 and 12.)

$$\begin{aligned} = \, & {} W_0 + W^{c_d}_{MGDL^*_{bd}}({\mathcal {C}}) + c_d(N-k). \end{aligned}$$

Note that $W_0$ does not depend on the topology of the species tree. Hence, the $(n-1)$-clique ${\mathcal {C}}$ with minimum weight defines a tree *ST* that minimizes $DL^*_{bd}(\mathcal {G}, ST,c_d)$. The proof for (b) follows trivially. $\square$

### Dynamic programming algorithm

Let $\mathcal {SBP}$ be a set of subtree-bipartitions, with $\mathcal {SBP}$ equal to all possible subtree-bipartitions if an exact solution is desired, and otherwise a proper subset if a faster algorithm is desired or necessary. We present the DP algorithm for the $MGDL^*_{bd}(\mathcal {G},c_d)$ problem. We compute *score*(*A*) in order, from the smallest cluster to the largest cluster $\mathcal {X}.$

- **Algorithm** $$MGDL^*_{bd}(\mathcal {G},c_d)$$
- if |A| = 1 then* score*(*A*) = *W*_*XL*_(*A*)
- else
- *score*(*A*) = *max*{*score*(*A*_1_) + *score*(*A* − *A*_1_) + $W_{MGDL^*_{bd}}^{c_d}$ (*A*_1_|*A* − *A*_1_) : (*A*_1_|A−*A*_1_) ∈ $\mathcal {SBP}\}$

If there is no $(A_1|A-A_1) \in \mathcal {SBP}$, we set *score*(*A*) to $-\infty$, signifying that *A* cannot be further resolved. At the end of the algorithm, if $\mathcal {SBP}$ includes at least one clique of size $n-1$, we have computed $score(\mathcal {X})$ as well as sufficient information to construct the optimal set of compatible clusters and hence the optimal species tree (subject to the constraint that all the subtree bipartitions in the output tree are in $\mathcal {SBP}$). If subtree bipartitions in $\mathcal {SBP}$ are not sufficient for building a fully resolved tree on $\mathcal {X}$, then $score(\mathcal {X})$ will be $-\infty$, and our algorithm returns FAIL.

The optimal number of duplications and losses is given by $score(\mathcal {X}) + c_d(N - k)$, by Theorem 11. If $\mathcal {SBP}$ contains all possible subtree-bipartitions, we have an exact but exponential time algorithm. However, if $\mathcal {SBP}$ contains only those subtree-bipartitions from the input gene trees, then the algorithm finds the optimal constrained species tree in time that is polynomial in the number of gene trees and taxa.

#### **Theorem 12**

*Let* $\mathcal {G}$ *be a set of rooted binary gene trees,* $\mathcal {SBP}$ *a set of subtree-bipartitions which contains only the subtree-bipartitions, or a subset of the subtree-bipartitions from the input gene trees. The dynamic programming (DP) algorithm finds the species tree ST minimizing the weighted duploss score, treating incomplete gene trees as resulting from gene birth and death, subject to the constraint that* $\mathcal {SBP}_{ST} \subseteq \mathcal {SBP}$ *in* $O(n|\mathcal {SBP}|^2)$ *time. Therefore, if* $\mathcal {SBP}$ *is all possible subtree-bipartitions, we have an exact but exponential time algorithm. However, if* $\mathcal {SBP}$ *contains only those subtree-bipartitions from the input gene trees, then the DP algorithm finds the optimal constrained species tree in* $O(d^2 n^3k^2)$ *time, where n is the number of species, k is the number of gene trees, and d the maximum number of times that any taxon appears in any gene tree.*

#### *Proof*

The proof of correctness is given above. The running time analysis for an arbitrary $\mathcal {SBP}$ follows the same argument as given in [20], since $W_{xl}(v)$ and $W_{dom}(v)$ can be computed in *O*(1) time after the preprocessing (as described in [20]). Finally, suppose *Q* is the set of subtree bipartitions from the input gene trees, and we use *Q* as the constraint set. Note that |*Q*| is *O*(*dkn*) (every internal node in every gene tree corresponds to subtree bipartition, and there are at most a total of *dkn* internal nodes across all the gene trees). Hence, when the constraint set is just the set *Q* of subtree bipartitions from the input set of gene trees, then the algorithm runs in $O(n|Q|^2) = O(d^2 k^2 n^3)$ time. $\square$

### Extensions

It is trivial to extend the theory for $MGDL^*_{bd}$ and $MGL^*_{bd}$ to $MGDL_{bd}$ and $MGL_{bd}$, as we now show. Recall that the only difference between $L^*_{bd}(gt,ST)$ and $L_{bd}(gt,ST)$ is whether the gene is assumed to be present at the ancestral species: in $L_{bd}(gt,ST)$ it is not assumed to be present there, but in $L_{bd}(gt,ST)$ it is. Therefore, $L_{bd}(gt,ST) = L^*_{bd}(gt,ST) - |UMMC(gt,ST)|$ and that $DL_{bd}(gt,ST) = DL^*_{bd}(gt,ST) - |UMMC(gt,ST)|$. Therefore, to extend the algorithmic approach to solve $MGL_{bd}$ and $MGDL_{bd}$, we define $W_{MGL_{bd}}(v,gt) = W_{MGL^*_{bd}}(v,gt)-W_{MMC}(v,gt)$ and $W_{MGDL_{bd}}(v,gt) = W_{MGDL^*_{bd}}(v,gt)-W_{MMC}(v,gt),$ and then seek a minimum weight maximum clique in the compatibility graph with these modified weights.

## Conclusion

The calculation of reconciliation costs between gene trees and species trees is a standard step in many bioinformatics analyses, including the estimation of species trees from a set of gene trees. This paper showed that different interpretations of incompleteness (i.e., species missing from genes) can impact the way that these reconciliation costs should be calculated, and need to be taken into account when using Gene Tree Parsimony to construct species trees from gene trees.

To address this issue, we presented a dynamic programming algorithm that provably finds an optimal species tree given a set of gene trees under the (weighted) GDL model within a constrained search space, treating incompleteness as due to true biological loss. This technique can be used on any input on which other gene tree parsimony is used. The use of dynamic programming to find provably optimal solutions within a constrained search space is also how ASTRAL (a coalescent-based species tree estimation method) [29–31] and FastRFS (a supertree method) [32] achieve good performance. For those methods, the constraints are based on bipartitions rather than subtree bipartitions, but the dynamic programming algorithm is nearly identical to the one we use here. As noted in [32], although setting the constraint set to just the bipartitions in the input source trees produced good results, expanding the set to include bipartitions from computed supertrees improved the topological accuracy of the resultant FastRFS supertree, without greatly increasing the running time. This suggests that expanding the subtree bipartition set to include estimated species trees based on GDL would be similarly beneficial for the dynamic programming method we present in this paper. In addition, changing the technique for defining subtree bipartitions is necessary when all the gene trees are incomplete, since in that case none of the gene trees can contribute any valid subtree bipartitions.

The results and the methods presented here are based on the assumption that the gene trees are discordant due to gene duplication and loss. However, these methods can be applied to both orthologous genes (in which case the gene trees could be single copy) and gene families that by definition will include both paralogs and orthologs. In cases where the genes are expected to be orthologs, all discordance between gene trees and species trees should be due to processes other than duplication and loss (e.g., incomplete lineage sorting), which could make approaches that attempt to minimize the total GDL cost potentially less accurate. Nevertheless, it is also possible that these GDL-based methods could be reasonably accurate under conditions where ILS rather than GDL is operating, and so these methods should be explored in that context.

Another natural source of discordance is gene tree estimation error, which is likely to occur with most biological datasets (see discussion in [33]). Therefore, the most accurate estimations of the number of duplications and losses (or weighted versions of these numbers) will only be obtained when the estimated gene trees and species trees are highly accurate, so that every attempt should be made to estimate these trees carefully. Gene trees, especially of multi-copy genes spanning large numbers of species, can be extremely large (i.e., greater than 100,000 leaves), and thus present enormous analytical and computational challenges (e.g., multiple sequence alignment and likelihood-based tree estimation are both difficult for datasets with more than about 1000 sequences, let alone 100,000) [34, 35]. Since completely accurate gene trees are not likely to be reliably obtained, these reconciliation methods and associated species tree estimation methods should be modified to take gene tree uncertainty into account. Methods such as NOTUNG [36] and ProfileNJ [37] are examples of methods that do this, but more work is needed.
